# Supplementary material for: Trends and determinants of the triple burden of malnutrition in Ghana; Analyses of two decades of demographic and health survey datasets
Source: PLOS Glob Public Health. 2025 Sep 12;5(9):e0005078. doi: 10.1371/journal.pgph.0005078 (PMC12431433; doi:10.1371/journal.pgph.0005078)
Supplement: Attachment — Submitted filename: Reviewer s Comments June 2025.docx [file pgph.0005078.s001.docx]

**Reviewer’s Comments for Manuscript Number PGPH-D-25-00980**

**Title:** It reflect all concepts in the findings

**Abstract:** well written and it provides all necessary summary information of findings

**Introduction:** Having gone through the literature and read some references provided, I can say, it is well thought and written.

**Methods:** The approach is well explained and illustrated

**Results:** Although I am not meticulously competent on statistics, the findings are very clear with approached statistics applied for providing the unknown information that some are easily also summarized by proving charts. My personal reflection is that results are well stipulated to possibly satisfies the readers to understand the key massages. Support information added value to the manuscript.

**Discussion and conclusion:** Well thought /written with comparison to the existing referenced literature on triple burden malnutrition and convincing way forward provided.
