# Supplementary material for: Trends and determinants of the triple burden of malnutrition in Ghana; Analyses of two decades of demographic and health survey datasets
Source: PLOS Glob Public Health. 2025 Sep 12;5(9):e0005078. doi: 10.1371/journal.pgph.0005078 (PMC12431433; doi:10.1371/journal.pgph.0005078)
Supplement: Attachment — Submitted filename: Geoff_reviewer 111.docx [file pgph.0005078.s002.docx]

**Introduction section**

1. The manuscript discusses undernutrition, micronutrient and overnutrition as distinct components of TBM. Is there evidence of a *direct or indirect relationship* between these two forms of malnutrition? For instance, could early-life undernutrition predispose individuals to obesity later in life?

The introduction will benefit if the authors explore these issues from literature

2. The manuscript presents TBM as the co-occurrence of undernutrition, overnutrition, and micronutrient deficiencies. However, if these conditions are interrelated (e.g., one leading to another), should TBM be reframed as a *systemic consequence* of shared underlying factors (e.g., food systems, socioeconomic disparities) rather than a standalone classification? What evidence supports or challenges this perspective?
3. If TBM is indeed a consequence of interconnected malnutrition forms, how should this shape interventions? For example, would integrate programs targeting shared drivers (e.g., food quality, inequity) be more effective than siloed approaches addressing undernutrition, obesity, or micronutrient deficits separately?

**Analysis section**

4. The manuscript defines TBM households as those with at least one form of malnutrition (undernutrition, overnutrition, or micronutrient deficiency). However, how were *non-TBM households* classified? Were they defined as households with *zero* forms of malnutrition, or was there another criterion (e.g., absence of all three)? Clarifying this distinction is critical for interpreting comparisons between TBM and non-TBM groups.
5. TBM was analyzed as a *binary categorical variable* (presence/absence). Given that malnutrition burdens can vary in severity or combination (e.g., 1, 2, or all 3 forms), was the consideration of treating TBM as:
   - An *ordinal variable* (e.g., 0, 1, 2, or 3 malnutrition types) to capture gradations of burden?
   - A *continuous composite score* (e.g., based on severity or frequency)?

What are the statistical or conceptual justifications for the chosen approach?

For instance, does a binary classification mask meaningful heterogeneity in risk factors or outcomes?

6. The use of logistic regression implies that the outcome (TBM) is modeled as a binary outcome. Were alternative models (e.g., multinomial logistic regression for >2 categories, or Poisson regression for count data) explored?

If TBM is a pooled of multiple malnutrition forms, could the analysis have been strengthened by modeling each component *separately* (e.g., using hierarchical or structural equation models) to identify unique drivers?
