# Supplementary material for: Trends and determinants of the triple burden of malnutrition in Ghana; Analyses of two decades of demographic and health survey datasets
Source: PLOS Glob Public Health. 2025 Sep 12;5(9):e0005078. doi: 10.1371/journal.pgph.0005078 (PMC12431433; doi:10.1371/journal.pgph.0005078)
Supplement: Attachment — Submitted filename: Response to reviewers.docx [file pgph.0005078.s005.docx]

**The Editor**

**PLOS Global Public Health**

**31^st^ July 2025**

**Dear Editor,**

**AUTHORS’ RESPONSES TO REVIEWERS’ COMMENTS/QUERIES**

The authors are grateful to you and the reviewers for your time and commitment to improving our manuscript titled “**Trends and determinants of the triple burden of malnutrition in Ghana; analyses of two decades of Demographic and Health Survey datasets**” (manuscript number PGPH-D-25-00980). The authors have edited the manuscript to improve it as suggested by you and the reviewers. In the text that follows, we respond to each of the comments/queries with references to the line numbers from the manuscript where the changes can be found.

**Journal Requirements:**
1. Please provide separate figure files in .tif or .eps format.
For more information about figure files please see our guidelines:
https://journals.plos.org/globalpublichealth/s/figures
https://journals.plos.org/globalpublichealth/s/figures#loc-file-requirements

2. Please review your reference list to ensure that it is complete and correct. If you have cited papers that have been retracted, please include the rationale for doing so in the manuscript text, or remove these references and replace them with relevant current references. Any changes to the reference list should be mentioned in the rebuttal letter that accompanies your revised manuscript. If you need to cite a retracted article, indicate the article’s retracted status in the References list and also include a citation and full reference for the retraction notice.

**Authors’ response**

1. Separate figure files have been provided in .tif format
2. No such retracted article(s) have been included in the reference list.

**Reviewer #1:**

**Reviewer #1 comment 1**

Trends and determinants of the triple burden of malnutrition in Ghana; analyses of two decades of Demographic and Health Survey datasets

Line 73: “As a result, the concurrent existence of maternal micronutrient deficiencies and obesity in an individual could burden health systems and retard progress in achieving nations’ set health and developmental targets.” Consider omitting one of the bolded words as it sounds a bit redundant.

**Response to reviewer comment 1**

The word “concurrent existence” has been corrected to “existence”. Please check line 75.

**Reviewer #1 comment 2**

Line 76: “The co-existence of malnutrition also has a potential to cloud the judgement of public health experts as to which malnutrition type deserves urgent attention within the context of scarce resources, particularly in the presence of undernutrition and overnutrition.” Consider omitting, its redundant

**Response to reviewer comment 2**

The sentence has been removed.

**Reviewer #1 comment 3**

Line 79: “While LMICs are more likely to be disproportionately affected by the TBM due to the pronounced effects of a rapid nutrition transition, and more recently the adverse effects of climate change, there is paucity of literature on the factors that drive TBM at the household, maternal and child-level in Ghana”
Climate change, as it currently stands in the sentence, seems forced because there is no mention of macro level factors (household, maternal and child-level only), so consider omitting or rephrasing

**Response to reviewer comment 3**

Right. The sentence has been rephrased to exclude “and more recently the adverse effects of climate change”. Please check lines 78 to 80.

**Reviewer #1 comment 4**

Line 93: “As a comparatively new concept, that lacks scholarship within previous maternal and child malnutrition discourse in Ghana, it is important to ascertain the trend of TBM over the past two decades.”
First half of this sentence needs rephrasing.

**Response to reviewer comment 4**

The first part of the sentence has been rephrased and now reads.

“As a comparatively new concept, with limited scholarship within previous maternal and child malnutrition discourse in Ghana, it is important to ascertain the trend of TBM over the past two decades” Please check lines 91 to 93.

**Reviewer #1 comment 5**
Line 184: “Likewise, an underweight child and a woman who is underweight and anaemic in the same household could also be classified as a household with TBM.”
This does not satisfy the TBD, as both mom and child here are undernourished. There is no overnutrition in this scenario, so it qualifies as a double burden.

**Response to reviewer comment 5**

Thanks for this input. The sentence has been corrected to include an “… woman who is overweight …”. Please check line 183.

**Reviewer #1 comment 6**
Line 206: “maternal education, religion, ethnicity”
Categories need to be mentioned, as done with the other variables.

**Response to reviewer comment 6**

The various categories of maternal education have been mentioned in line 207. However, religion and ethnicity have been expunged because they remain redundant throughout subsequent sections of the manuscript.

**Reviewer #1 comment 7**
Line 210: “This was calculated based on 24 hr-food consumption patterns from the FAO & FHI 10-food group classifications [35]; 1) grains, white roots and tubers, and plantains, 2) beans, peas and lentils, 3) nuts and seeds 4) milk and milk products 5) meat, poultry and fish, 6) eggs, 7) dark green leafy vegetables 8) other vitamin A rich fruits and vegetables, 9) other vegetables and 10) other fruits.”
For standardization purposes, since the different food groups were mentioned for women’s diet diversity score, the same needs to be done for the child’s different food groups earlier.

**Response to reviewer comment 7**

Right. The different food groups for children’s dietary diversity have been included. Please check lines 194 to 196.

**Reviewer #1 comment 8**
Line 281: “74.7% of children aged < 24 months were still breastfeeding,”
Why wasn’t exclusively breast fed further considered? Given the WHO recommendations and the critical role it plays in malnutrition.
**Response to reviewer comment 8**

There were two factors that determined which variables were considered for inclusion in this study. 1) its inclusion in previous TBM literature and 2) its presence in DHS datasets. None of the TBM articles measured exclusive breastfeeding as a standalone variable; partly because if such a variable was measured it would be strongly correlated with still breastfeeding variable since the former is a subset of the latter. Also, the inclusion of breastfeeding status as a part of the dietary diversity variable for some datasets (2022) and not for other datasets (2003, 2008 and 2014) made it more difficult to identify exclusive breastfeeding as a standalone variable for the purposes of this study.

**Reviewer #1 comment 9**
Line 307: “Mothers overweight had a pooled prevalence of 35.2% (34.2 - 26.2).”
For standardization purposes, why hasn’t the trend for overweight mentioned as well? (up from x).

**Response to reviewer comment 9**

The trend for overweight has already been stated in line 309.

**Reviewer #1 comment 10**
Line 407: “Where malnourished children are more likely to suffer overweight and overweight-related non-communicable diseases later in life due to early-life undernutrition”
This is not a sentence, it needs revision.

**Response to reviewer comment 10**

The sentence has been corrected. Please check lines 409 to 411.

**Reviewer #2:**

The manuscript tackles a relevant topic but lacks novelty in its main claims. Several assertions are made without proper citation, and the literature review is thin, failing to place the study adequately within existing research. The analysis is shallow, with limited interpretation of regression results and no control for key confounding variables. Lastly, the discussion includes claims that are not fully grounded in the presented data. A major revision is needed to address issues of depth, referencing, and transparency.

**Response to Reviewer # 2 comments**

The Authors are of the view that the entire manuscript could be termed as novel in its approaches as well as its claims (findings) because this is the first country-specific manuscript on TBM that used data spanning 2 decades. It is equally noteworthy to state that this Journal (PLOS Global Public Health) places more weight on Scientific contribution/relevance rather than novelty in determining if a manuscript is fit for publication. To the best of our knowledge, the Authors have provided proper citations for all assertions, placed this study within the context of existing literature in Ghana and elsewhere, conducted rigorous statistical analyses and the results well interpretated.

We are thankful to the reviewer for his efforts at trying to constructively critique this manuscript. If the reviewer could be more specific in some of their comments on Authors not being “transparent” or even pinpoint aspects of the discussions that are not “grounded in the presented data” that would have helped us improve this manuscript.

**Reviewer #3 comments**

I congratulates all who participated in preparing the manuscript. To me the manuscript is well thought, written and it provide new experience and insight in reviewing data sets of the long existing data sets of DHS programs not only in Ghana, but also it may attract more researchers and academia in different countries to look into their existing data. You have shown a way to others

**Response to Reviewer # 3 comments**

We are thankful to the reviewer for the positive comments.

**Reviewer #4 comments**

The article was well written and cogent. However, the article will benefit from some areas that were not explored i,e interrelationship between the different types of malnutrition, analysis of TBM as outcome of complex issues. The statistical analysis section will benefit if authors could explain choice of how variables were use ( form of utilization ) and the exact choice of models used .

**Response to Reviewer # 4 comments**

Specific changes have been carried-out on lines 71 to 74 to reflect the fact that one aspect of TBM are linked to another. For the fact that TBM is an outcome of complex issues, the definition of TBM has been expanded on lines 53 to 55 to reflect this.

The sentence on line 152 has also been amended to reflect how variables were entered into the multivariable model.

The following statement has been added on the statistical test used to assess model significance.

*“The chi-square value and overall model p-value of the Wald test were used to assess model significance.”* Please check lines 260 and 261.

**Geoff Review comments (Reviewer #5)**

**Introduction section**

1. The manuscript discusses undernutrition, micronutrient and overnutrition as distinct components of TBM. Is there evidence of a *direct or indirect relationship* between these two forms of malnutrition? For instance, could early-life undernutrition predispose individuals to obesity later in life?

The introduction will benefit if the authors explore these issues from literature.

**Authors response to reviewer #5 comment 1**

Rightly so. There is direct evidence that links early-life undernutrition/overweight with deleterious health outcomes later in life. For instance, in the introduction lines 71 to 73 talks about undernutrition and impaired cognitive development and the chances of childhood overweight and increasing the risks of chronic non-communicable diseases later in life.

However, the specific relationship of how “*early-life undernutrition predisposes individuals to obesity later in life*” as suggested by the reviewer, has been explored in the discussion section lines 405 to 411.

The authors discussed a major finding of this study (increasing overweight/obesity rates) by postulating that early-life undernutrition could account for the increasing maternal obesity rates (in the discussion section). Therefore, it might appear repetitive if same is incorporated into the introduction section.

**Reviewer #5 comment 2**

2. The manuscript presents TBM as the co-occurrence of undernutrition, overnutrition, and micronutrient deficiencies. However, if these conditions are interrelated (e.g., one leading to another), should TBM be reframed as a *systemic consequence* of shared underlying factors (e.g., food systems, socioeconomic disparities) rather than a standalone classification? What evidence supports or challenges this perspective?

**Authors’ response to comment 2**

The authors agree that the individual conditions of TBM could be complex and interrelated as espoused in response to the reviewer’s first comment. Therefore, TBM has been reframed on lines 53 to 55 of the introduction as below.

*“This is commonly known as the triple burden of malnutrition (TBM) [3,4],* *a systemic consequence of shared underlying factors emanating from food systems inadequacies and socioeconomic disparities*.”

**Reviewer #5 comment 3**

3. If TBM is indeed a consequence of interconnected malnutrition forms, how should this shape interventions? For example, would integrate programs targeting shared drivers (e.g., food quality, inequity) be more effective than siloed approaches addressing undernutrition, obesity, or micronutrient deficits separately?

**Authors’ response to comment 3**

The authors are thankful for this suggestion. The authors have added the below statement to lines 502 to 504 of the discussion.

*“**… interventions with integrated programs targeting shared drivers (e.g., food quality, inequity) may be more effective than siloed approaches addressing undernutrition, obesity, or micronutrient deficits separately.”*

**Reviewer #5 comment 4**

**Analysis section**

4. The manuscript defines TBM households as those with at least one form of malnutrition (undernutrition, overnutrition, or micronutrient deficiency). However, how were *non-TBM households* classified? Were they defined as households with *zero* forms of malnutrition, or was there another criterion (e.g., absence of all three)? Clarifying this distinction is critical for interpreting comparisons between TBM and non-TBM groups.

**Authors’ Response to comment 4**

The manuscript defined TBM households as those with all three forms of malnutrition in their respective combinations - undernutrition (child or maternal undernutrition), overnutrition (child or maternal overnutrition), and micronutrient deficiency (child or maternal iron deficiency aneamia).

Based on the above, a household could be termed non TBM if it has zero forms of malnutrition, or has only one form of malnutrition or 2 forms of malnutrition, i.e. only undernutrition, only overnutrition or only micronutrient deficiency. Malnutrition can exist without the concurrent presence of all three forms; and that is what this study is interested in.

**Reviewer #5 comment 5**

5. TBM was analyzed as a *binary categorical variable* (presence/absence). Given that malnutrition burdens can vary in severity or combination (e.g., 1, 2, or all 3 forms), was the consideration of treating TBM as:
   - An *ordinal variable* (e.g., 0, 1, 2, or 3 malnutrition types) to capture gradations of burden?
   - A *continuous composite score* (e.g., based on severity or frequency)?

What are the statistical or conceptual justifications for the chosen approach?

For instance, does a binary classification mask meaningful heterogeneity in risk factors or outcomes?

**Authors’ response to comment 5**

Yes, TBM was analyzed as a binary categorical outcome variable. It is true that malnutrition burdens can vary in severity based on respective combinations, e.g. 1 form of malnutrition, 2 forms of malnutrition and all 3 forms carry different burdens. It should however, be noted that the co-occurrence of all 3 forms of malnutrition is the focus of this present manuscript.

The presence of 1 form of malnutrition and 2 forms (double burden of malnutrition) has been extensively explored in published literature within the Ghanaian context. As such, the objective of this manuscript was to focus specifically on the occurrence of all 3 forms of malnutrition in the same household. And not the respective graduation of the different combinations of malnutrition.

The conceptual justification for combining malnutrition in the form it was done in this study emanates from the 2020 UNICEF conceptual framework for maternal and child nutrition. In this publication, UNICEF states, “*The 2020 Framework builds on UNICEF’s 1990 conceptual work, acknowledges the increasing triple burden of malnutrition – undernutrition, micronutrient deficiencies, and overweight – and highlights the role of diets and care as immediate determinants of maternal and child nutrition.*”

The statistical justification comes from the various published articles on TBM that used a similar approach to combine these 3 types of malnutrition into a single composite TBM. These articles are cited on line 162.

It does mask heterogeneity emanating from the extraneous graduations of the different forms of malnutrition (stated earlier). The binary outcome method therefore isolates only TBM which is the main outcome of interest in this study.

**Reviewer #5 comment 6**

6. a. The use of logistic regression implies that the outcome (TBM) is modeled as a binary outcome. Were alternative models (e.g., multinomial logistic regression for >2 categories, or Poisson regression for count data) explored?

b. If TBM is a pooled of multiple malnutrition forms, could the analysis have been strengthened by modeling each component *separately* (e.g., using hierarchical or structural equation models) to identify unique drivers?

**Authors’ response to comment 6**

a. TBM was classified as binary; as such both methods stated above may not be applicable given the study’s objective.

b. We acknowledge that the triple burden of malnutrition represents a composite indicator that integrates multiple, and often biologically distinct forms of malnutrition. Hence, modelling each component separately could indeed help to uncover unique determinants specific to each malnutrition form. However, as stated earlier, this study emphasizes on TBM only. The authors risk derailing from the original intent of this study should we go ahead with additional models. This response is provided in view of the fact that there are numerous published articles in Ghana that have modelled the determinants the individual components of TBM i.e. undernutrition, overnutrition and micronutrient deficiencies.

**Comments from ‘reviewer #6’**

**Reviewer’s Comments for Manuscript Number PGPH-D-25-00980**

**Title:** It reflect all concepts in the findings

**Abstract:** well written and it provides all necessary summary information of findings

**Introduction:** Having gone through the literature and read some references provided, I can say, it is well thought and written.

**Methods:** The approach is well explained and illustrated

**Results:** Although I am not meticulously competent on statistics, the findings are very clear with approached statistics applied for providing the unknown information that some are easily also summarized by proving charts. My personal reflection is that results are well stipulated to possibly satisfies the readers to understand the key massages. Support information added value to the manuscript.

**Discussion and conclusion:** Well thought /written with comparison to the existing referenced literature on triple burden malnutrition and convincing way forward provided.

**Authors’ response to reviewer #6 comments**

Thank you for this positive review.

**PDF Annotations**

1. How will the TBM cloud the judgement of public health experts as to which malnutrition deserves urgent attention?
2. Is there relationship between undernutrition and over nutrition. What about hidden hunger? If there is, then TBM is not an occurrence its self but a consequence of the individual types of malnutrition.
3. So how was a non TBM defined? A household which will have at least one form of malnutrition, a household with two or non.
4. Why was TBM treated as a categorical variable but not ordinal or continuous variable
5. Explain why the use of logistic regression.

**Authors’ Responses to** **PDF Annotations**

1. The statement has been expunged.
2. In response to a previous comment, a statement has been added to buttress the relationship between the various components of TBM. Please check line …
3. A household with none, one and two forms of malnutrition will be described as non TBM.
4. The intent was to isolate only households with TBM (undernutrition, overnutrition and micronutrient deficiency) so as to satisfy the objective of this study.
5. We used **logistic regression** because our main dependent variable—the triple burden of malnutrition (TBM)—was constructed as a **binary outcome**, indicating whether or not a child experienced any form of malnutrition as stated above. Logistic regression may be the most suitable modelling approach for binary outcomes, as it estimates the **odds of the outcome occurring** based on a set of predictor variables. This approach is well-established in epidemiological and health research and has previously been used in other studies – Chilot et al, 2023 (doi:10.1136/bmjopen-2022-070978) and Kumar et al., 2021 (doi:10.1186/s12889-021-10411-w)

**Concluding remarks**

The authors appreciate the depth of this review. We have presented responses/explanations to the reviewers’ comments/queries. Hopefully, this should be enough to make this manuscript publication worthy in your most respected Journal.

Sincerely Yours

*Signed*

Addae Yaw Hammond

**Corresponding Author**
